# Water Deficit-Induced Changes in Phenolic Acid Content in Maize Leaves Is Associated with Altered Expression of Cinnamate 4-Hydroxylase and p-Coumaric Acid 3-Hydroxylase

**DOI:** 10.3390/plants12010101

**Published:** 2022-12-25

**Authors:** Zintle Kolo, Anelisa Majola, Kyle Phillips, Ali Elnaeim Elbasheir Ali, Robert E. Sharp, Ndiko Ludidi

**Affiliations:** 1 Department of Biotechnology, University of the Western Cape, Robert Sobukwe Road, Bellville 7530, South Africa; 2 2-8 Agriculture Building, Interdisciplinary Plant Group, Division of Plant Science and Technology, University of Missouri, Columbia, MO 65211, USA; 3 DSI-NRF Centre of Excellence in Food Security, University of the Western Cape, Robert Sobukwe Road, Bellville 7530, South Africa

**Keywords:** water deficit, drought stress, phenolic acids, cinnamate 4-hydroxylase, *p*-coumarate 3-hydroxylase

## Abstract

The amino acid phenylalanine is a precursor to phenolic acids that constitute the lignin biosynthetic pathway. Although there is evidence of a role of some phenolic acids in plant responses to pathogens and salinity, characterization of the involvement of phenolic acids in plant responses to drought is limited. Drought reduces water content in plant tissue and can lead to decreased cell viability and increased cell death. We thus subjected maize seedlings to water deficit and evaluated relative water content and cell viability together with p-coumaric acid, caffeic acid and ferulic acid contents in the leaves. Furthermore, we measured the enzymatic activity of cinnamate 4-hydroxylase (EC 1.14.13.11) and p-coumarate 3-hydroxylase (EC 1.14.17.2) and associated these with the expression of genes encoding cinnamate 4-hydroxylase and p-coumarate-3 hydroxylase in response to water deficit. Water deficit reduced relative water content and cell viability in maize leaves. This corresponded with decreased p-coumaric acid but increased caffeic and ferulic acid content in the leaves. Changes in the phenolic acid content of the maize leaves were associated with increased enzymatic activities of cinnamate 4-hydroxylase and p-coumarate hydroxylase. The increased enzymatic activity of p-coumarate 3-hydroxylase was associated with increased expression of a gene encoding p-coumarate 3-hydroxylase. We thus conclude that metabolic pathways involving phenolic acids may contribute to the regulation of drought responses in maize, and we propose that further work to elucidate this regulation may contribute to the development of new maize varieties with improved drought tolerance. This can be achieved by marker-assisted selection to select maize lines with high levels of expression of genes encoding cinnamate 4-hydroxylase and/or p-coumarate 3-hydroxylase for use in breeding programs aimed and improving drought tolerance, or by overexpression of these genes via genetic engineering to confer drought tolerance.

## 1. Introduction

Maize (*Zea mays* L.), a staple food in many countries and an important livestock feed crop, is generally not tolerant to the water deficit that occurs during drought [1]. Drought is a widespread phenomenon globally and its occurrence is expected to be more frequent, longer and more intense due to climate change [2]. This necessitates the development of maize lines with improved tolerance to drought. To achieve this goal, it is necessary to understand how components of various metabolic pathways in maize are influenced by stresses such as drought because the perturbation of these pathways impacts maize crop survival and yield [3].

Phenolic acids occur naturally in plants [4,5,6] and perform a wide variety of physiological functions underscored by their roles as signaling molecules [7] and in defense [8] and antioxidant activities [9]. Measurements limited to total phenolic acid content rather than the content of specific phenolic acids show that phenolic acids are involved in plant responses to water deficit stress [10]. To date, the changes in specific phenolic acid content in response to water deficit have been reported only in a few plant species including *Amaranthus tricolor* [11] without investigating the relationship between such changes and the expression of genes or activities of enzymes associated with phenolic acid biosynthesis.

Drought induces excessive production of reactive oxygen species (ROS), which have to be scavenged by antioxidant enzymes and non-enzymatic antioxidants [12]. Recently, an enzyme with dual activity as both a p-coumarate 3-hydroxylase (C3H, EC 1.14.17.2) and an ascorbate peroxidase (APX, EC 1.11.1.11) has been identified in *Brachypodium distachyon* and *Zea mays* [13], highlighting association of phenolic acid metabolism with ROS metabolism. Given the link between drought, ROS and antioxidant activities of phenolic acids, it is relevant to establish how phenolic acid content is influenced by drought. Changes in phenolic acid content during development of seeds in different varieties of wheat are associated with changes in the expression of genes involved in phenolic acid biosynthesis [14]. Phenolic acid biosynthesis initiates with the conversion of phenylalanine by phenylalanine-ammonia lyase (EC 4.3.1.5) to produce cinnamic acid, which is converted to p-coumaric acid via a reaction catalyzed by cinnamate 4-hydroxylase (C4H, EC 1.14.13.11) [15,16]. An alternative pathway produces p-coumaric acid via conversion of tyrosine to tyrosine-ammonia lyase [17]. The p-coumaric acid can be metabolized to produce caffeic acid through a downstream reaction catalyzed by p-coumarate 3-hydroxylase, with caffeic acid serving as a precursor for the biosynthesis of ferulic acid in a reaction catalyzed by hydroxycinnamaldehyde dehydrogenase (HCALDH, EC 1.2.1.68) or caffeic acid/5-hydroxy ferulic acid-O-methyltransferase (COMT, EC 2.1.1.68) [6].

Given the various important roles of phenolic acids in plant biology, which include responses to stress [18], we determined the influence of water deficit on the levels of a subset of free phenolic acids in maize leaves; namely p-coumaric acid, caffeic acid and ferulic acid. To account for developmentally regulated responses, and in addition to well-watered (WW) temporal controls, we included well-watered plants that were at the same stage of development as the plants subjected to water deficit. Furthermore, we measured the activities of cinnamate 4-hydroxylase and p-coumarate 3-hydroxylase since these enzymes are involved in the biosynthesis of the phenolic acids that we analyzed. We also assessed the expression of a gene encoding p-coumarate 3-hydroxylase to establish if a relationship exists between phenolic acid content, phenolic acid biosynthesis enzymes and phenolic acid biosynthesis genes.

## 2. Results

**Figure 1:**
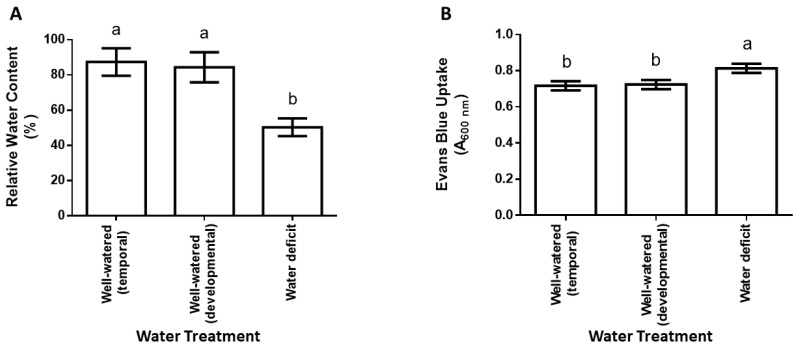
Effect of water deficit on relative water content (**A**) and cell death (**B**) in maize leaves. The third youngest leaf from three different plants in each treatment (well-watered temporal and developmental controls, water deficit) was used. Data are means ± SD from three independent experiments, where different letters above error bars indicate means that are significantly different (*p* ≤ 0.05).

Water deficit decreased the relative water content by approximately 0.4-fold (Figure 1A). The reduced relative water content in the water deficit (WD) treatments was accompanied by a marginal decrease in cell viability, reflected by an approximately 0.1-fold increase in Evans blue uptake in the water deficit treatment (Figure 1B).

**Figure 2:**
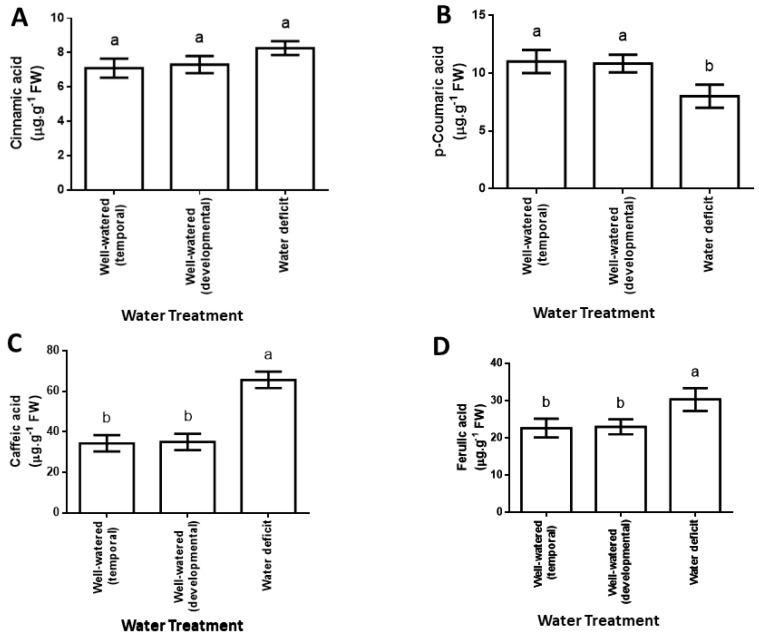
Effect of water deficit on cinnamic acid (**A**), p−coumaric acid (**B**), caffeic acid (**C**) and ferulic acid (**D**) contents in maize leaves. Data represent means from results obtained in extracts of the third youngest leaves from three independent experiments. FW represents fresh weight. Error bars with different letters represent significantly different means ± SD at a confidence level defined by *p* ≤ 0.05.

Water deficit did not have a significant effect on cinnamic acid levels in maize leaves but resulted in a 0.3-fold decrease in leaf *p*-coumaric acid content, whereas it caused a 0.9-fold increase in leaf caffeic acid content and a 0.3-fold increase in ferulic acid content (Figure 2A–D).

**Figure 3:**
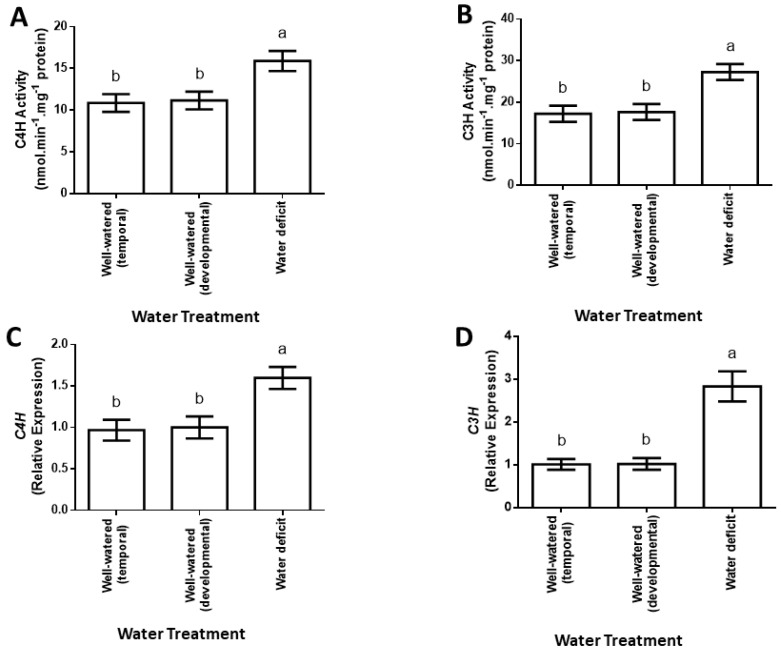
Changes in the enzymatic activities of C4H (**A**) and C3H (**B**) in relation to effects of water deficit on the expression of genes encoding a cinnamate 4−hydroxylase (**C**) and a p−coumarate 3−hydroxylase (**D**) in maize leaves. Data represent means of three independent experiments in tissue obtained from the third youngest leaf of each treatment. C4H is cinnamate 4−hydroxylase and C3H is p−coumarate 3−hydroxylase. Error bars with different letters represent significantly different means ± SD at a confidence level defined by *p* ≤ 0.05.

On the one hand, decreased p-coumaric acid content (Figure 2B) was associated with a 0.4-fold increase in C4H enzymatic activity (the enzyme participating in the biosynthesis of p-coumaric acid; Figure 3A). On the other hand, increased caffeic acid content (Figure 2C) was associated with a 0.6-fold increase in C3H enzymatic activity (the enzyme participating in the biosynthesis of caffeic acid; Figure 3B). The level of the phenolic acids and enzymatic activities described herein did not differ significantly between the leaves of well-watered temporal and developmental control plants (Figure 2 and Figure 3A,B).

Upon observing differences in *p*-coumaric acid and caffeic acid contents and enzymatic activity for C4H and C3H, we investigated if water deficit alters the expression of the maize genes encoding C4H and C3H. Use of the 2^−ΔΔT^ method in the qPCR data to calculate transcript accumulation showed a 0.6-fold increase in C4H gene expression in response to water deficit (Figure 3C), whereas C3H gene expression increased 1.8-fold in the leaves in response to the water deficit treatment (Figure 3D).

Water deficit caused significant changes (*p* ≤ 0.05) in all the assessed parameters except for the amount of cinnamic acid except (Table 1). The highest change was in the level of expression of p-coumarate 3-hydroxylase and the associated metabolite, namely caffeic acid (Table 1).

## 3. Discussion and Conclusions

The decrease of relative water content in leaves of plants subjected to water deficit in the work reported here, taken together with reduced cell viability in these leaves, indicates that drought stress causes a water loss-related decrease in cell viability, which may be a consequence of drought-induced physiological changes that lead to cell death in the leaves. A decrease in leaf p-coumaric acid levels in response to drought has previously been reported for leaves of *Ligustrum vulgare* grown under full sunlight [19] and *Vitis vinifera* in response to drought [20], which are similar observations to those reported here. However, there are also reports where no significant change in p-coumaric acid content occurred (for example, in leaves of rice) under drought stress [21], whereas p-coumaric acid content in *Amaranthus tricolor* leaves increased in response to drought [11]. The increase in caffeic acid and ferulic acid content in the maize leaves observed in our study agrees with other studies showing a similar trend in leaves of plants subjected to drought stress [19], but contradicts the trend seen in *Vitis vinifera* in the case of caffeic acid [20]. It is important to note that the drought stress treatments in these experiments on different plant species differed in intensity and method of application. It is thus likely that the varying response trends in relation to phenolic acid levels in response to drought is species dependent and influenced by the level of water deficit stress experienced by the plants. 

Notwithstanding the similarities and contradictions in the various reports in respect of the changes in phenolic acid levels in response to drought, the results reported here clearly show that water deficit in maize leads to altered phenolic acid metabolism in the case of *p*-coumaric acid, caffeic acid and ferulic acid. Important to note are the effects of water deficit on the activities of enzymes involved in phenolic acid biosynthesis. The observed augmentation of C4H activity in response to water deficit, when considered together with the resulting decrease in p-coumaric acid, implies that drought acts to shift phenolic acid metabolism towards increased biosynthesis of phenolic acids. On the one hand, this could possibly provide antioxidative phenolics to reduce the oxidative stress that would result from drought-triggered excessive accumulation of ROS, given the involvement of ROS in drought responses [12]. On the other hand, it could be a means to contribute phenolics which are required for cell wall lignification as a measure to maintain turgor under water deficit, since drought is known to cause cell wall lignification for this purpose [22]. If this were true, and in light of the fact that C4H acts to convert cinnamic acid to *p*-coumaric acid [15,16], increased C4H activity should result in increased p-coumaric acid content. However, this was not the case in the work reported here since a decrease in *p*-coumaric acid content is observed instead. This is not surprising though, because the increase in C4H activity occurred together with pronounced increase in C3H activity (Figure 3A,B).

Given that C3H functions to produce caffeic acid at the expense of *p*-coumaric acid [6,18], it is argued here that the augmented C3H activity in response to drought consumes the *p*-coumaric acid produced by the C4H activity and this results in lowered *p*-coumaric acid levels despite the increased C4H activity. The consequence of the substantially increased C3H activity is reflected in the large increase we observed in the level of its catalytic product, namely caffeic acid. The elevated caffeic acid pool would serve as a source of substrate for COMT and/or HCALDH [6,18] for the biosynthesis of ferulic acid, resulting in the observed increase in ferulic acid in response to drought.

Increased enzymatic activity for C4H and C3H could be due to posttranslational modification of the protein, once synthesized. Alternatively, it could be a consequence of increase in the amount of protein synthesized due to increased expression of the mRNA encoding the protein. The observed increase in the expression of the genes encoding C4H and C3H, as seen here from qPCR analyses, is supportive of a transcript-driven accumulation of C4H and C3H even though this does not exclude post-transcriptional and post-translational modifications that would impact enzymatic activity.

From the results observed here, it can be concluded that drought induces enzymatic activity of C4H and C3H, which may be driven, in part, by increased expression of genes such as those encoding C4H and C3H. These drought-induced changes would lead to elevated levels of phenolic acids such as caffeic acid and ferulic acid. The changes in the phenolic acid contents may impact plant antioxidant activity due to the role of phenolic acids such as caffeic acid in antioxidant activity [10,11]. This phenolic acid-mediated response may contribute to the regulation of maize responses to drought and thus serves as an avenue for exploring engineering of the phenylpropanoid pathway to alter phenolic acid biosynthesis for improvement drought tolerance in maize. To this end, marker-assisted selection to select maize lines with high levels of expression of genes encoding cinnamate 4-hydroxylase and/or p-coumarate 3-hydroxylase can be used in breeding programs towards improvement of drought tolerance. An alternative approach to achieve such trait improvement is the overexpression of these genes using genetic engineering to confer drought tolerance.

## 4. Materials and Methods

### 4.1. Reagents and Plant Materials

All chemicals were analytical or molecular grade and were purchased from Sigma-Aldrich (St. Louis, MO, USA) unless stated otherwise. Maize [*Zea mays* (L.) cv. Border King] seeds were purchased from a commercial seed company, McDonalds Seeds (Mkondeni, Pietermaritzburg, South Africa). Promix Organic was purchased from Windell Hydropoics CC (Kraaifontein, Cape Town, South Africa). Nitrosol plant nutrient concentrate and Wonder Iron Chelate were purchased from Efekto Care Pty Ltd. (Benmore, Johannesburg, South Africa). Perspex tubes were purchased from Maizey Plastics (Stikland, Bellville, South Africa). 

### 4.2. Methods

#### 4.2.1. Plant Growth and Water Deficit Treatments

Maize seeds were surface-sterilized using chlorine gas by following a procedure described by Paz et al. [23]. For this sterilization, seeds were placed in one layer in standard Petri dishes in a desiccator jar inside a fume hood. NaClO (100 mL of a 12% solution) was mixed with 4 mL of concentrated HCl in a 250 mL beaker in the fume hood. The beaker was placed in the desiccator jar where the seeds in open Petri dishes were also placed. The desiccator jar was tightly closed immediately and left in the fume hood for 16 h. Seeds were removed from the Petri dishes and placed on a moist paper towel in plastic containers and covered with lids. The containers with the seeds were placed in the dark at 4 °C for 4 days. The containers were transferred to a dark cabinet at 23 °C and seeds were allowed to germinate and grow in the containers until radicles were 0.5–1 cm long.

Promix Organic (Windell Hydroponics CC, Cape Town, South Africa) was supplemented with nutrients by adding Nitrosol [Efekto Care Pty (Ltd.), Johannesburg, South Africa] and Iron Chelate [Efekto Care Pty (Ltd.), Johannesburg, South Africa] at thrice the concentrations recommended by the manufacturers. The water potential of the Promix Organic was adjusted with water to −0.03 MPa for growing plants under well-watered temporal or developmental control conditions and −0.45 MPa for growing plants under water deficit conditions at the time of transplanting, on the basis of measurements taken using a WP4C Water Potential Meter (Campbell Scientific, Logan, UT, USA). Germinated seeds (10 seeds per Perspex tube) with radicles approximately 0.5 cm (not exceeding 1 cm) in length were transplanted 3 cm deep into 28 L of the Promix Organic contained in green Perspex tubes (1 m tall, 20 cm diameter) which were placed on plastic pot trays. The tubes were placed in a greenhouse programmed to have light supplied by high pressure sodium lamps (600 W) for 16 h (day time from 05:00 until night time from 21:00) with a minimum temperature of 18 °C (night) and a maximum temperature of 28 °C (day).

Seedlings in all three treatments were supplemented once a week for 2 weeks by watering the Promix Organic with 100 mL of nutrient solution containing a mixture of Nitrosol and Iron Chelate according to the instructions from the manufacturers. The solution was added to the plastic trays in which the Perspex tubes were placed. Thereafter, for well-watered control conditions a further 400 mL of tap water was added once a week, whereas for the water deficit treatment, the Promix Organic was allowed to dry down over the course of the experiment without any further watering; at the time of harvesting, the water potential was approximately −0.75 ± 0.08 MPa at a depth of approximately 15 cm. Developmental control plants and water deficit plants were harvested at the V5 stage of vegetative growth (4 weeks after transplanting into Promix Organic for developmental control and 6 weeks for water deficit plants), whereas well-watered temporal control plants were harvested at the V11 stage of vegetative growth (6 weeks after transplanting into Promix Organic).

#### 4.2.2. Measurement of Relative Water Content

The third youngest leaf from three different plants in each treatment (well-watered temporal and developmental controls, water deficit) was used. A 3 cm long section (from the base) of the leaf was harvested at midday and the fresh weight was immediately recorded. The leaf section was placed in a 50 mL centrifuge tube filled with distilled water for 16 h in the dark, after which the leaf section was blotted dry on paper towel to remove surface water and weighed to obtain the turgid weight. The leaf was then dried in an oven at 70 °C for 48 h, placed in a desiccator filled with dry silica and rapidly weighed to obtain the dry weight. The weights were used to calculate leaf relative water content according to the formula described by Downey and Miller [24] where relative water content = (fresh weight − dry weight)/(turgid weight − dry weight).

#### 4.2.3. Measurement of Cell Viability

Cell viability was measured in a 4 cm^2^ section (from the base) from the third youngest leaf from three different plants in each treatment according to a modified method described by Sanevas et al. [25]. The leaf material was incubated in 1 mL of 0.25% (*w*/*v*) Evans Blue stain in a microcentrifuge tube for 15 min at room temperature. The stain was removed and leaf sections were washed three times in 1 mL of deionized water for 30 min each time. After removal of the water, Evans Blue taken up by the cells of the leaf tissue was extracted by incubating the leaf tissue in 1 mL of 1% (*w*/*v*) SDS at 55 °C for 1 h. The samples were settled by centrifugation at 3000× *g* for 1 min at room temperature. The supernatant was transferred to a clean microcentrifuge tube and 200 µL of the supernatant was read for absorbance at 600 nm, with 200 µL of 1% SDS used as a blank whose absorbance was subtracted from the sample absorbance to obtain the value used to calculate cell viability. The resulting difference was divided by the weight of the 4 cm^2^ leaf section and expressed as Evans Blue uptake to indicate the extent of cell death in the sample.

#### 4.2.4. Measurement of Free Phenolic Acids

The third youngest leaf from three different plants in each treatment was used to measure p-coumaric acid, caffeic acid and ferulic acid content. For these measurements, 100 mg of freshly weighed leaf tissue from each sample was extracted by grinding the leaf tissue in 1 mL of 70% methanol/water (*v*/*v*). This was followed by addition of 100 μL of 100 nM naphthol as an internal standard. The mixture was briefly vortexed and incubated for 120 min at 60 °C. The samples were centrifuged and 500 µL of the supernatant from each sample were transferred to clean microcentrifuge tubes. The supernatants were dried in a SpeedVac^TM^ SPD120 vacuum drier (Thermo Scientific, Waltham, MA, USA) at 42 °C. When samples were dry, they were reconstituted with 150 µL acetonitrile followed by addition of 50 µL N, O-Bis(trimethylsilyl)trifluoroacetamide made in 1% trimethylsilyl chloride. The mixture was vortexed and then derivatized by incubating for 1 h in an oven maintained at 80 °C. After incubation, the mixture was again vortexed and then transferred into a gas chromatography vial with an insert. The samples (1 µL each) were injected on a Thermo Scientific TSQ 8000 MS quadrupole operated in Selective Reaction Monitoring (SRM) mode.

Separation of the heterocyclic amines was performed on a Thermo Scientific TRACE^TM^ 1310 gas chromatograph coupled with a non-polar (95% dimethylpolysiloxane) capillary column (Restek-Rxi^®^-5Sil MS w/Intrega-Guard^®^; 15 m, 0.25 mm internal diameter, 0.25 µm film thickness). The initial oven temperature was 100 °C and was held for 4 min, then subsequently increased at 6 °C/min to 180 °C, then held for 2 min with a final temperature of 250 °C at a rate of 15 °C/min, followed by a final hold time of 5 min. The injector and transfer line temperatures were maintained at 250 °C and 280 °C, respectively. Helium at a 1 mL/min flow rate was used as the carrier gas. The ionization source temperature was set at 250 °C and emission current of 50 µAmperes was used with argon collision. The concentrations of p-coumaric acid, caffeic acid and ferulic acid were calculated against corresponding standards of known concentration subjected to the same gas chromatographic measurements.

#### 4.2.5. Measurement of C4H and C3H Activity

Enzymatic activities associated with phenolic acid biosynthesis, namely C4H for p-coumaric acid biosynthesis and C3H for caffeic acid biosynthesis, were measured in extracts from the third youngest leaf of three different plants from each treatment.

C4H activity was measured based on a method modified from Jadhav et al. [26] using 200 mg of freshly harvested leaf tissue from each treatment. The tissue was ground into a fine powder in liquid nitrogen and homogenized in 600 µL of 100 mM Tris buffer (pH 7.5) and centrifuged at 3000× *g* for 20 min at 4 °C. The supernatants were transferred to clean microcentrifuge tubes and 50 µL of the supernatant was added to a reaction mixture of a total volume of 200 µL containing 200 µM cinnamic acid, 500 µM NADPH, 1 mM EDTA at pH 7.5 and 50 mM phosphate buffer, pH 7.4, the reaction of which was allowed to proceed at 30 °C for 5 min. The reaction was stopped by adding 20 µL of concentrated glacial acetic acid. Absorbance of the reaction was read on a FLUOstar microplate reader (BMG Labtech, Ortenberg, Germany) at 340 nm and the enzymatic activity for C4H was calculated using an extinction coefficient of 6200 M^−1^ cm^−1^ for *p*-coumaric acid accumulation.

For C3H activity, a procedure modified from Jadhav et al. [26] was used in which 200 mg of freshly harvested leaf tissue from each treatment was ground into a fine powder in liquid nitrogen in the presence of 20 mg polyvinylpolypyrrolidone, followed by homogenization in 800 µL of 50 mM phosphate buffer (pH 7.4) that had been equilibrated to 4 °C by storage in a refrigerator. The mixture was centrifuged for 15 min at 10,000× *g* at 4 °C. The reaction was started by taking 50 µL of the resulting supernatant and mixing it with 150 µL of reaction components consisting of 0.5 mM *p*-coumaric acid and 50 mM potassium phosphate, pH 7.0, which was then incubated in the dark for 1 h at 28 °C. After this step, 60 μL of concentrated glacial acetic acid was added to stop the reaction, followed by centrifugation of the mixture at 13,000× *g* for 30 min at 4 °C. After this step, 200 µL of the supernatant from each sample was transferred to a 96-well clear plate and absorbance values were read at 325 nm using a FLUOstar microplate reader. Absorbance values were used to calculate enzymatic activity based on an extinction coefficient of 15,700 M^−1^ cm^−1^ for caffeic acid production.

#### 4.2.6. Analysis of Cinnamate 4-Hydroxylase and p-Coumarate 3-Hydroxylate Gene Expression

The expression of the genes encoding cinnamate 4-hydroxylase (C4H) and p-coumarate 3-hydroxylate (C3H) was assessed in total RNA extracts from the third youngest leaf of three different plants from each treatment. Total RNA was extracted using the Direct-Zol™ RNA miniprep kit (Zymo Research, Irvine, CA, USA) by following the instructions provided in the product guidelines. To eliminate DNA from the RNA preparation, RNase-free DNase I (Zymo Research) was used as specified in the instruction sheet. This was followed by application of 2 µL of RiboLock^®^ RNase Inhibitor (Thermo Scientific) to each RNA sample to prevent degradation of the RNA. From each total RNA sample isolated above, first strand cDNA was synthesized using 500 ng of total RNA with the RevertAid™ Reverse Transcriptase kit (Thermo Scientific) according to the instructions from the manufacturer, with a random hexamer primer (supplied with the kit) used in the cDNA synthesis.

Gene-specific primers (Primer sequences in Table 2) were used to amplify the C4H and C3H genes from 2 µL of each sample of synthesized first strand cDNA, with the maize actin-2, β-tubulin and Elongation Factor 1 α genes (Primer sequences in Table 1) as internal control genes for quantitative PCR.

For the quantitative PCR, first strand cDNA from the above experiments was used on a Luminaris Color HiGreen™ Low ROX qPCR master mix (Thermo Scientific) as described by the manufacturer. Reactions were run on a Roche LightCycler^®^ 480 system (Penzberg, Germany) following manufacturer guidelines. Primer sequences for the internal control genes are given in Table 1. Gene expression levels of C4H and C3H in the various treatments were presented as ratios relative to the values of the well-watered samples, based on the average accumulation of the transcripts of the internal control genes using the 2^−ΔΔT^ method [27].

#### 4.2.7. Statistical Analysis

Data from three independent experiments described above were subjected to one-way analysis of variance (ANOVA) and assessed for significance using the Tukey–Kramer test at a 5% level of significance with the aid of GraphPad Prism 6.0.1.

## Figures and Tables

**Table 1:** Comparison of means of measured physiological, biochemical and molecular changes in maize grown in well-watered (temporal), well-watered (developmental) or water deficit conditions.

	RWC	Evans Blue Uptake	Cinnamic Acid	p-Coumaric Acid	Caffeic Acid	Ferulic Acid	C4H Activity	C3H Activity	C4H Expression	C3H Expression
Well-watered (temporal)	87.33 ± 6.34 a	0.72 ± 0.02 b	7.10 ± 2.05 a	11.00 ± 0.82 a	34.33 ± 3.30 b	22.67 ± 2.05 b	10.87 ± 0.86 b	17.23 ± 1.60 b	0.97 ± 0.10 b	1.01 ± 0.10 b
Well-watered (development)	84.33 ± 6.94 a	0.72 ± 0.02 b	7.30 ± 1.63 a	10.83 ± 0.62 a	35.00 ± 3.27 b	23.00 ± 1.63 b	11.17 ± 0.86 b	17.67 ± 1.55 b	1.00 ± 0.11 b	1.02 ± 0.11 b
Water deficit	50.33 ± 4.11 b	0.81 ± 0.02 a	8.27 ± 2.49 a	8.00 ± 0.82 b	65.67 ± 3.30 a	30.33 ± 2.49 a	15.90 ± 0.99 a	27.27 ± 1.55 a	1.60 ± 0.11 a	2.83 ± 0.29 a
F	24.06	13.81	4.843	9.903	59.26	8.593	19.51	26.15	22.15	62.87
*p*	0.001	0.006	0.056	0.013	0.000	0.017	0.002	0.001	0.002	0.000

Abbreviations are for relative water content (RWC), cinnamate 4−hydroxylase (C4H) and p−coumarate 3−hydroxylase (C3H). Data presented are means ± SD; n = 3. Means with different letters are significantly different at *p* ≤ 0.05.

**Table 2:** Sequences of primers used as oligonucleotides for analyses of gene expression in maize leaves in response to water deficit.

Primer Name	Sequence (5′–3′)
C3H_1F	TCA TCT CCG TCT GGT TCG GG
C3H_1R	AGC CTC CTG GGC GTG AAG A
C4H_1F	GCG TAA GAA GGT GAT GGC T
C4H_1R	AGG AGG TTG TCG TGG TTG AT
Act2F	CTGAGGTTCTATTCCAGCCATCC
Act2R	CCACCACTGAGGACAACATTACC
β-tubF	CTACCTCACGGCATCTGCTATGT
β-tubR	GTCACACACACTCGACTTCACG
EF1αF	GGGCCTACTGGTCTTACTACTGA
EF1αR	ACATACCCACGCTTCAGATCCT

## Data Availability

All data are available upon request.

## References

[1] Meng Q., Chen X., Lobell D.B., Cui Z., Zhang Y., Yang H., Zhang F. (2016). Growing sensitivity of maize to water scarcity under climate change. Sci. Rep..

[2] Lobell D.B., Roberts M.J., Schlenker W., Braun N., Little B.B., Rejesus R.M., Hammer G.L. (2014). Greater sensitivity to drought accompanies maize yield increase in the U.S. Midwest. Science.

[3] Sun C., Gao X., Fu J., Zhou J., Wu X. (2014). Metabolic response of maize (*Zea mays* L.) plants to combined drought and salt stress. Plant Soil.

[4] Acosta-Estrada B.A., Gutiérrez-Uribe J.A., Serna-Saldívar S.O. (2014). Bound phenolics in foods, a review. Food Chem..

[5] Adom K.K., Liu R.H. (2002). Antioxidant activity of grains. J. Agric. Food Chem..

[6] Nair R.B., Bastress K.L., Ruegger M.O., Denault J.W., Chapple C. (2004). The *Arabidopsis thaliana* REDUCED EPIDERMAL FLUORESCENCE1 gene encodes an aldehyde dehydrogenase involved in ferulic acid and sinapic acid biosynthesis. Plant Cell.

[7] Mandal S.M., Chakraborty D., Dey S. (2010). Phenolic acids act as signaling molecules in plant-microbe symbioses. Plant Signal Behav..

[8] Bhattacharya A., Sood P., Citovsky V. (2010). The roles of plant phenolics in defence and communication during Agrobacterium and Rhizobium infection. Mol. Plant Pathol..

[9] Piazzon A., Vrhovsek U., Masuero D., Mattivi F., Mandoj F., Nardini M. (2012). Antioxidant activity of phenolic acids and their metabolites: Synthesis and antioxidant properties of the sulfate derivatives of ferulic and caffeic acids and of the acyl glucuronide of ferulic acid. J. Agric. Food Chem..

[10] Puente-Garza C.A., Meza-Miranda C., Ochoa-Martínez D., García-Lara S. (2017). Effect of in vitro drought stress on phenolic acids, flavonols, saponins, and antioxidant activity in *Agave salmiana*. Plant Physiol. Biochem..

[11] Sarker U., Oba S. (2018). Drought stress enhances nutritional and bioactive compounds, phenolic acids and antioxidant capacity of *Amaranthus* leafy vegetable. BMC Plant Biol..

[12] Laxa M., Liebthal M., Telman W., Chibani K., Dietz K.J. (2019). The role of the plant antioxidant system in drought tolerance. Antioxidants.

[13] Barros J., Escamilla-Trevino L., Song L., Rao X., Serrani-Yarce J.C., Palacios M.D., Engle N., Choudhury F.K., Tschaplinski T.J., Venables B.J. (2019). 4-Coumarate 3-hydroxylase in the lignin biosynthesis pathway is a cytosolic ascorbate peroxidase. Nat. Commun..

[14] Ma D., Li Y., Zhang J., Wang C., Qin H., Ding H., Xie Y., Guo T. (2016). Accumulation of phenolic compounds and expression profiles of phenolic acid biosynthesis-related genes in developing grains of white, purple, and red wheat. Front. Plant Sci..

[15] Schoch G.A., Nikov G.N., Alworth W.L., Werck-Reichhart D. (2002). Chemical inactivation of the cinnamate 4-hydroxylase allows for the accumulation of salicylic acid in elicited cells. Plant Physiol..

[16] Pierrel M.A., Batard Y., Kazmaier M., Mignotte-Vieux C., Durst F., Werck-Reichhart D. (1994). Catalytic properties of the plant cytochrome P450 CYP73 expressed in yeast. Substrate specificity of a cinnamate hydroxylase. Eur. J. Biochem..

[17] Rösler J., Krekel F., Amrhein N., Schmid J. (1997). Maize phenylalanine ammonia-lyase has tyrosine ammonia-lyase activity. Plant Physiol..

[18] Dixon R.A., Paiva N.L. (1995). Stress-induced phenylpropanoid metabolism. Plant Cell.

[19] Tattini M., Galardi C., Pinelli P., Massai R., Remorin D., Agat G. (2004). Differential accumulation of flavonoids and hydroxycinnamates in leaves of *Ligustrum vulgare* under excess light and drought stress. New Phytol..

[20] Król A., Amarowicz R., Weidner S. (2014). Changes in the composition of phenolic compounds and antioxidant properties of grapevine roots and leaves (*Vitis vinifera* L.) under continuous of long-term drought stress. Acta Physiol. Plant..

[21] Quan N.T., Anh L.H., Khang D.T., Tuyen P.T., Toan N.P., Minh T.N., Minh L.T., Bach D.T., Ha P.T.T., Elzaawely A.A. (2016). Involvement of secondary metabolites in response to drought stress of rice (*Oryza sativa* L.). Agriculture.

[22] Le Gall H., Philippe F., Domon J.-M., Gillet F., Pelloux J., Rayon C. (2015). Cell wall metabolism in response to abiotic stress. Plants.

[23] Paz M.M., Shou H., Guo Z., Zhang Z., Banerjee A.K., Wang K. (2004). Assessment of conditions affecting Agrobacterium-mediated soybean transformation using the cotyledonary node explant. Euphytica.

[24] Downey L.A., Miller J.W. (1971). Rapid measurements of relative turgidity in maize (*Zea mays* L.). New Phytol..

[25] Sanevas N., Sunohara Y., Matsumoto H. (2007). Characterization of reactive oxygen species-involved oxidative damage in *Hapalosiphon* species crude extract-treated wheat and onion roots. Weed Biol. Manag..

[26] Jadhav P.R., Mahatma M.K., Jha S., Mahatma L., Parekh V.B., Jha S.K. (2013). Changes in phenylpropanoid pathway during compatible and incompatible interaction of *Ricinus communis-Fusarium oxysporum* f.sp. ricini. Ind. J. Agric. Biochem..

[27] Livak K.J., Schmittgen T.D. (2001). Analysis of relative gene expression data using real-time quantitative PCR and the 2(-Delta Delta C(T)) Method. Methods.

